# Co-barcoded sequence reads from long DNA fragments: a cost-effective solution for “perfect genome” sequencing

**DOI:** 10.3389/fgene.2014.00466

**Published:** 2015-01-14

**Authors:** Brock A. Peters, Jia Liu, Radoje Drmanac

**Affiliations:** ^1^Department of Research, Complete Genomics Inc., Mountain ViewCA, USA; ^2^BGI, ShenzhenChina

**Keywords:** WGS, whole genome sequencing, haplotyping, NGS, MPS, “perfect genome, ” LFR, *de novo* assembly

## Abstract

Next generation sequencing (NGS) technologies, primarily based on massively parallel sequencing, have touched and radically changed almost all aspects of research worldwide. These technologies have allowed for the rapid analysis, to date, of the genomes of more than 2,000 different species. In humans, NGS has arguably had the largest impact. Over 100,000 genomes of individual humans (based on various estimates) have been sequenced allowing for deep insights into what makes individuals and families unique and what causes disease in each of us. Despite all of this progress, the current state of the art in sequence technology is far from generating a “perfect genome” sequence and much remains to be understood in the biology of human and other organisms’ genomes. In the article that follows, we outline why the “perfect genome” in humans is important, what is lacking from current human whole genome sequences, and a potential strategy for achieving the “perfect genome” in a cost effective manner.

## INTRODUCTION

Next generation sequencing (NGS) technologies, primarily based on massively parallel sequencing (MPS; Drmanac and Crkvenjakov, 1990; Shendure et al., 2005; Bentley et al., 2008; Drmanac et al., 2010), have touched and radically changed almost all aspects of research worldwide. These technologies have allowed for the rapid analysis, to date, of the genomes of more than 2,000 different species (http://www.genomesonline.org/statistics). In humans, NGS has arguably had the largest impact. Over 100,000 genomes of individual humans (based on various estimates) have been sequenced allowing for deep insights into what makes individuals and families unique and what causes disease in each of us (Rios et al., 2010; Roach et al., 2010; Kong et al., 2012; MacArthur et al., 2012; Michaelson et al., 2012; O’Roak et al., 2012; Sanders et al., 2012; Tennessen et al., 2012; Veltman and Brunner, 2012; Epi et al., 2013; Schaaf et al., 2013; Yang et al., 2013; Al Turki et al., 2014; Fromer et al., 2014; Gilissen et al., 2014; Lawrence et al., 2014; Purcell et al., 2014).

Massively parallel sequencing technologies continue to advance in efficiency with recent improvements allowing “single pixel per spot” imaging on patterned DNA arrays to generate many terabases of read data per sequencing run (Peters et al., 2012) and the recent introduction of Illumina’s HiSeq X, also using patterned arrays, producing over 500 Gb per day. By our estimates, a further several-fold increase in MPS efficiency will be achieved in the next few years, including a reduction in the cost to approximately $1/Gb of raw reads. Despite all of this progress, the current state of the art in sequence technology is far from perfect and much remains to be understood in the biology of human and other organisms’ genomes. Further, before large-scale adoption of whole genome sequencing (WGS) in humans as a standard of clinical practice can be realized, the quality along multiple different metrics must improve several fold to several orders of magnitude. These improvements can potentially lead to practically complete and errorless reading of individual human genomes. In the article that follows, we outline why this “perfect” WGS (“perfect genome”) in humans is important, what is lacking from current human whole genome sequences, and a potential strategy for achieving the “perfect genome” in a cost-effective manner.

## THE BENEFITS OF BEING PERFECT

Within the approximately three billion DNA letters inherited from each parent and the sixteen thousand maternally inherited mitochondrial letters (about 6 billion total) exists a program for the development and adaptive functioning of all of our tissues. As such, it is reasonable to believe that perfect analysis of the human genome will become the ultimate genetic test (Green et al., 2011; Drmanac, 2012) for the discovery of all medically and otherwise relevant variants. Moreover, DNA sequencing of the inherited genome can be administered once and utilized for the entire life of an individual. At the level of a single individual cell there can be many changes in the genome, but averaged over millions of cells in multiple tissues, and excluding special circumstances such as mosaicism or other events that can cause a few dominant clonal genomes, there is little change in a person’s genome over a life time. There are important exceptions to this, such as the detection of somatic mutations causing diseases such as cancer where periodic genome sequencing would need to be done on tissue biopsies or blood samples. Coupling perfect WGS with a complete database of genomic variants and gene networks and their corresponding phenotypes will allow for a powerful human health prediction and disease prevention tool. The importance of perfect WGS is analogous to the obvious benefits of errorless reading of computer-stored human-generated information. Error-prone retrieval of data files would have greatly limited the value of information technologies and significantly impacted their wide use in every day life. Because nuclear and mitochondrial genomes comprise the complete genetic information, perfect sequencing of millions of human genomes per year will form a new irreplaceable infrastructure (similar to the Internet infrastructure) for growing many industries (health, pharmaceuticals, food, sports, and education) with the ultimate goals of improving and extending human life.

## WHAT IS PERFECT?

Perfect WGS or the “perfect genome” is free of errors, meaning there is no need to validate medically relevant variants using some orthogonal sequencing method. DNA bases are not skipped nor are additional bases added in a perfectly read genome and we can be confident that everything that could affect the health of the individual, including all *de novo* mutations, has been found. In perfect WGS, all deletions, duplications, and disease-causing repeat expansions are detected and the length of each of the 92 telomeres and 24 centromeres is accurately measured. Perfect WGS requires the separate assembly of each parental chromosome (i.e., full haplotype phasing) allowing for the context of all genomic variations to be understood. Contextual interpretation of the influence of genomic variants upon each other is critical for complete understanding of disease predisposition. Even a low false positive or negative error rate would impede any extensive contextual interpretation of genomic variants. Furthermore, a perfectly read genome allows for the full understanding of the complex interactions of genetic and environmental factors. Finally, perfect WGS must be cost effective, ideally less than 1,000 US dollars, otherwise it will be too expensive to be broadly utilized. In practice, it may be difficult to ever achieve a truly “perfect genome,” but a genome with a few errors and some unresolved repeat sequences is very obtainable and for the purposes of improving human health, it is exactly what is required. This practically “perfect genome” is what we refer to in the remainder of this article.

## FAR FROM PERFECT

### WHAT’S MISSING?

Over half of the human genome is composed of repetitive sequence elements (de Koning et al., 2011). The most popular and cost-effective sequencing technologies are currently limited to read lengths less than a few 100 base pairs and mate-pair distances typically less than several kilobases; those technologies with longer read lengths are currently prohibitively expensive for whole human genome sequencing or still have error rates too high for routine use. These read architectures allow for sequencing through many repeats, but some repetitive regions are still too long and thus unmappable. Approximately 5–10% of the human genome falls into these regions that are too long to be analyzed by current technologies. Additionally, some copy number variants (CNVs), especially extra copies of genomic regions <100 kb in length and genes with multiple functional or nonfunctional (e.g., pseudogenes) copies can be difficult to analyze accurately. To further complicate matters, current sequencing technologies struggle with regions of extreme AT or GC base content, some of which are important coding and regulatory sequences. Finally, many rare variants, such as *de novo* mutations, long insertions or deletions, and variants having low read coverage due to various biases are not detected due to the preference to call reference bases or known variants in a reference-based genome assembly.

### HUMANS ARE DIPLOID

Most individuals have more than 2 million locations in their genome where the 22 autosomal chromosomes inherited from each parent differ (Levy et al., 2007; Bentley et al., 2008; Wang et al., 2008; Wheeler et al., 2008; Ahn et al., 2009; Drmanac et al., 2010; Peters et al., 2012). These variants can have profound effects on the biology of the individual depending on if they are found on the same chromosome or opposite homologous chromosomes. Read lengths of a few hundred base pairs or less are insufficient to determine which combination of variants exists on a single parental chromosome. As a result most whole genome assemblies are haploid consensus in nature with both variants listed at each genomic position where a difference is found, but little to no information about the parental chromosome from which they came. For example, in the case where two detrimental variants are found in a single gene it is imperative to know if at least one of the parental genes is functional for genetic diagnosis. For imprinted genes a single detrimental variant can be disease-causing depending on the parental chromosome on which it resides (Schaaf et al., 2013).

### FINDING A SIGNAL IN THE NOISE

Next generation sequencing has begun to become more prevalent in the clinical setting, but false positive error rates are still too high [one false positive error in every 1 million bases (Roach et al., 2010)] for WGS to become routine clinical practice. False positive errors cloud the already difficult analysis of WGS data, creating potentially misleading detrimental variants that require additional analysis work or sequence validation to confirm. Many of these errors come from mismapping sequence reads between genomic regions evolved by duplication or repeat multiplication. Of equal importance are those variants that are missed in regions that are otherwise called or variants removed by filters during the genome assembly process to reduce false positive errors. All clinical tests suffer from these false positive and negative errors, however, WGS has much room to improve on these dimensions.

### LESS IS MORE

Thousands of cells are not always available in a clinical setting, but that is the amount of DNA required by most WGS technologies. Ideally WGS could be performed on 10 cells or less. This would allow for the application of WGS to *in vitro* fertilized embryo biopsies (Peters et al., under review *Genome Research*), circulating tumor cells, circulating or other fetal cells (Bolnick et al., 2014), and other biological samples with a small number of cells available. Starting with a small amount of DNA (66 pg in 10 human cells) requires DNA amplification that introduces false positive errors and other artifacts and biases making perfect WGS from a few cells especially challenging.

## TOWARD PERFECTION

**FIGURE 1:**
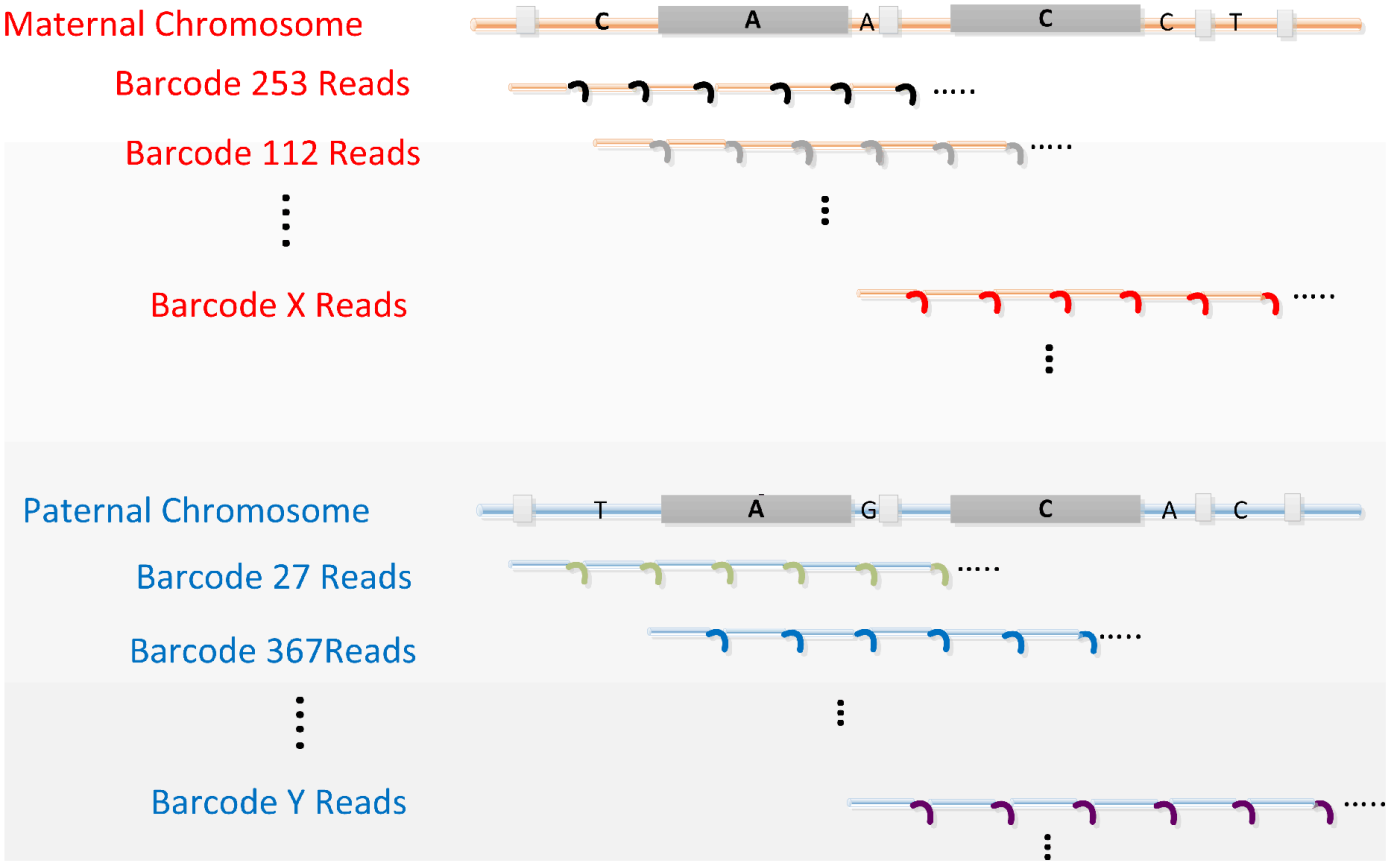
**The concept of read co-barcoding for advanced whole genome sequencing (WGS).** All four critical requirements are depicted. (1) A genomic library is prepared from long DNA (e.g., 30–300 kb) representing 10 or more cells. Multiple staggered long DNA fragments for each genomic region are generated as a result of random fragmenting during cell lysis (three fragments depicted under each parental chromosome). In the co-barcoded read libraries these redundant long fragments allow variant phasing, a more accurate assembly of the genome, and ultimately *de novo* assembly. In this example a pair of long proximate repeat elements, longer than the read and mate-pair length, is shown by the large gray boxes. A and C denote single base differences between copies of these repeat elements. Long, overlapping, staggered genomic fragments allow for the proper placement of these repeats in the final assembly by exclusive linking of repeat members to surrounding unique sequences provided by the long DNA fragments that start or end between repeats. (2) Sequence reads generated from each long fragment (i.e., subfragments used to produce these reads) are tagged (small colored curved lines) with the same barcode (co-barcoded). There are many (usually 10s–100s) of reads per long DNA fragment, most if not all having the same barcode. Reads belonging to related (i.e., overlapped) long fragments mostly have different barcodes. Consequently maternal (red) and paternal (blue) fragments for a genomic region have different barcodes as indicated by the distinct barcode numbers (253, 112, and X for mom, 27, 367, and Y for dad). After MPS, barcodes are used to aggregate reads from the original long fragment. Such read aggregation, even without sequence assembly per long fragment, provides information for variant phasing and repeat resolving when reads from overlapping long fragments, representing the same chromosome, are used together in the assembly process. (3) Sequence reads must cover >30% and preferably the majority of bases in each long fragment. Consecutive continuous reads (depicted here) or overlapping mate-pair reads (two shorter reads from the ends of the same subfragment) can provide the needed coverage. Sequencing the majority of bases of each fragment with co-barcoded reads links alleles in haplotypes as, on average, 10 or more heterozygous sites occur per long DNA fragment. (4) The read or mate-pair length is longer than the frequent dispersed repeats (e.g., Alu, depicted by the small gray boxes) and are correctly assembled primarily using read level data.

Our “perfect genome” solution employs advanced massively parallel DNA sequencing of “co-barcoded” reads from long genomic DNA molecules, and efficient *de novo* assembly empowered by these barcoded reads. The critical requirements of this fundamental and comprehensive solution for sequencing complex diploid genomes are depicted in **Figure 1**.

First, a sample providing sufficient (e.g., >10X) genome coverage in long (e.g., 30–300 kb) DNA fragments is needed. This redundancy allows the differentiation of true sequences from errors and artifacts and the separation of long clustered repeats (e.g., full length LINEs or segmental duplications) as discussed below. Implicit in this requirement is DNA isolated from at least several cells from one individual and the preservation of long DNA molecules. This eliminates formalin-fixed paraffin-embedded (FFPE) samples, in which DNA is typically degraded, and single cells as sources for our “perfect genome.”

Second, nearly all reads from a long fragment must share the same unique barcode (“co-barcoding”) and that barcode must be different from the barcodes used to tag reads from the majority of (i.e., >80%) related long DNA fragments (e.g., overlapped DNA fragments covering a genomic region or copies of long repeats). However, reads from many unrelated long fragments can have the same barcode.

Third, sufficient base coverage from each long DNA fragment (at least ∼30% and preferably >60% of bases with read coverage per fragment between 0.5X and 2X) is needed to link most of the sequence belonging to a long fragment and achieve almost all the benefits of very long reads (i.e., >50 kb). In the case of ideal unbiased WGS, a total read coverage of 40X would be sufficient for 10 diploid cells with co-barcoding, although in practice over 100X would be preferred.

Fourth, a read structure, as discussed below, that allows for the resolution of frequent short repeats (e.g., Alu and similar repeats) and the ability to measure the length of homopolymers and tandem repeats is important.

It is critical to satisfy all four listed requirements in this optimally designed arrangement in such a manner that DNA tagging does not add significantly to the total cost.

### DEMONSTRATION OF CO-BARCODED READS

The principles behind co-barcoded reads (**Figure 1**) have previously been described (Drmanac, 2006) and an experimental implementation of these principles called long fragment read technology (LFR; Peters et al., 2012) has been developed. Briefly, long related DNA fragments (e.g., overlapped fragments from a genomic region) are separated stochastically by pipetting a dilute high molecular weight DNA solution into physically distinct wells, followed by multiple displacement amplification (MDA; Dean et al., 2002) and fragmentation to generate shorter molecules (subfragments) suitable for short read mate-pair sequencing. Barcoded adapter molecules, unique to each well, are then ligated to the subfragments. Finally, the barcoded DNA from all wells is combined and sequenced, including reading the 10-base barcodes. All reactions preceding pooling are performed in the same plate without any DNA purification between steps enabling use of a large number of small wells. The initial dilution and separation of long DNA molecules is done such that approximately 10% of a haploid genome (∼300 Mb) is found in each well. Statistically this results in a 5% chance that for a given region of the genome overlapping molecules from each parent will be present in the same well. When performed in a 384-well plate there is a redundancy of ∼20 long fragments from each parent (∼40 in total), for any position in the genome, distributed across 36 wells on average. This results in each genomic location having DNA exclusively from one parent in 17 (2 of 36 wells have mixed parents) wells, on average, with an overall range of 6–20 wells.

### A LARGE NUMBER OF BARCODES IS CRITICAL FOR PERFECT WGS

Co-barcoding, as previously demonstrated (Peters et al., 2012), allows for haplotyping and substantial error correction, making it a key part of working toward the “perfect genome.” However, 384 wells do not allow for efficient *de novo* assembly and as will be discussed later, *de novo* assembly is absolutely critical for a “perfect genome.” The problem is that with 10% of the genome labeled by each barcode, and the repetitive nature of the human genome, there are too many overlapping short reads from distinct genomic regions in each well. The obvious way to improve this is to increase the number of physically distinct compartments and thus the number of barcodes, which reduces the percent of a haploid genome for each compartment.

How many barcodes are needed? The more the better, but to be able to measure common long repeats such as telomeres from 20 cells (∼2,000 telomeres) we would need at least 4,000 compartments (providing >75% isolated telomeres). This number of compartments is also needed to accurately count mitochondrial genomes and assess heteroplasmy as there are often more than 100 mitochondria per cell. Therefore, 5,000–10,000 is a good starting point. Using 10,000 individual compartments with 10,000 unique barcodes and 20 cells would result in only one hundred and twenty 100 kb molecules (∼12 Mb of DNA) per barcode. This small number of bases per each barcode dramatically reduces the chance of co-barcoding related long fragments. Additionally, 10,000 barcodes reduces the number of highly similar Alu repeats and LINEs with the same barcode to <1,000 for each repeat type. However, while 10,000 barcodes should be sufficient this suggests a larger number would be beneficial for analyzing these highly redundant DNA elements.

With more barcodes it may be more optimal to use more cells, ideally 50 if available, to increase the number of staggered long DNA fragments covering each genomic region. This would provide more power to separate long proximate repeats as discussed in **Figure 1**. More cells would also enable more independent measurements for error reduction (discussed below), assuming more reads are generated. For 1X read coverage reading ∼60% of bases of each long DNA molecule from 50 cells, 100X total read coverage is needed without any coverage bias. On average, this would result in 30 independent reads per parental base and cost about $300 to generate with the projected reduction of sequencing costs to $1/Gb.

### ELIMINATING LONG DNA AMPLIFICATION

Using 10,000 barcodes and 50 or more cells allows for the possibility to skip amplification of long DNA [e.g., MDA (Dean et al., 2002), MALBAC (Zong et al., 2012), or long range PCR]. Current long DNA amplification strategies introduce coverage bias and, if PCR is used (Kuleshov et al., 2014) limit fragment length to less than 10 kb. Ultimately, this amplification bias requires additional read coverage, 200X or more, to assemble a complete genome if there are no regions with severe under-amplification. Libraries barcoded prior to any amplification would result in non-overlapping adjacent co-barcoded 500–1,500 bp subfragments generated from each long DNA molecule. Using current MPS equipment would result in only the ends of each subfragment being read, and thus a lower fraction of bases per long fragment would be interrogated (∼30%). This would significantly complicate analysis and most likely would require improved read architecture as discussed later. Otherwise, minimal long DNA amplification of 10–100X may provide a good balance between the fraction of bases read per fragment and coverage bias.

### HOW LONG SHOULD LONG FRAGMENTS BE?

Long DNA fragments, over 1 Mb in some cases, are needed to phase across long genomic regions with no or a few heterozygous loci commonly found in the genomes of non-African populations (Peters et al., 2012) or to assess centromeric regions. However, for resolving intermediate repeats, such as LINEs, that are longer than mate-pair lengths random pools of one hundred approximately 100 kb fragments are more valuable than single 10 Mb fragments (**Figure 1**). An optimal solution is a broad distribution of fragment lengths from 30 kb to 3 Mb prepared from at least 50 cells, potentially using multiple different methods (e.g., enzymatic and mechanical) to improve the randomness of fragment ends. This would provide both frequent starts of fragments and a sufficient number of very long fragments (i.e., 3 Mb). This could be achieved by splitting cells into separate reactions prior to lysis and then performing different amounts of DNA fragmenting on each reaction. To preserve very long DNA, a reversible chromatin crosslinking process could potentially be used (de Vree et al., 2014).

### CAN WE USE 10,000 BARCODES COST-EFFECTIVELY?

Five thousand to 10,000 individual DNA aliquots is achievable with current technologies using our multi-step no-purification biochemical protocol (Peters et al., 2012) and there are companies (e.g., WaferGen, Fluidigm, etc.) selling devices that can dispense pico to nanoliters of liquid into microwells that can accommodate total volumes up to several hundred nanoliters. Alternatively, over 100,000 compartments can be generated easily in less than a minute using currently available emulsion based approaches (e.g., Raindrop, Bio Rad, etc.) and combinatorial barcoding in nanodrops (Drmanac et al., 2011). As these technologies improve the cost of using them will drop to become less than the cost of a 384-well plate. Importantly, if nanoliter or picoliter volume aliquots are used, the cost of reagents will be less than our current co-barcoding process (∼$50, several microliters per aliquot; Peters et al., 2012).

### WHAT READ STRUCTURE IS BEST?

Current MPS read lengths of about 50 bases or more are suitable for our proposed “perfect genome” strategy. However as discussed above, long DNA amplification will most likely be necessary if this read architecture is used. If an approach without long DNA amplification proves to be beneficial, a feasible near-term solution could be improved paired-end MPS with ∼300 base reads from each end of 500–1,500 bp subfragments (including the barcode sequence). Cost-effective and scalable single molecule sequencing of more than 500 bases would be even better if and when it becomes available. These longer paired-end or single reads could improve the resolution of repeats in close proximity to each other that are longer than current reads but too short to be resolved by the mate-pair read structure due to variation in the subfragment size (generally varying from 500 to 1,500 bp). Furthermore, longer reads reduce the negative effects of non-randomness from DNA fragmenting. 500 base reads would also provide a more precise measure of long triple and other tandem repeats. In our proposed co-barcoding based strategy, longer continuous reads (e.g., 5 kb) are less beneficial, especially if they are error prone and/or more expensive.

### ERROR FREE REFERENCE FREE PHASED GENOME ASSEMBLY

Our low cost “perfect genome” solution requires 10–100 cells and thus has to have a low read coverage per each long fragment (usually less than 2X), otherwise it would be very expensive to generate 600X read coverage (10 cells × 2 parental sets of chromosomes × 30X standard read coverage). Therefore, the trivial approach of collecting all reads with the same barcode to assemble long DNA fragments is not applicable. Instead, integrating low read coverage from 10 or more original long overlapped fragments with different barcodes per each genomic region is required. We previously described an approach for variant calling disregarding barcodes and then using barcodes for phasing heterozygous variants by calculating the number of shared compartments for alleles in the neighboring heterozygous loci (Peters et al., 2012). Using this method we also demonstrated that many false positive errors could be removed as it is unlikely for errors to repeatedly occur (i.e., have supporting reads with multiple different barcodes) exclusively on one parental chromosome, and if errors do occur on only one parental chromosome they are typically from a single barcoded fragment. In both cases, these errors can be identified and removed (Peters et al., 2012).

The above approaches are very effective but use a reference-biased process in the initial variant calling. Using a large number of barcodes allows implementation of a computationally and cost efficient (less than $200 by our estimates) reference-free or reference-unbiased assembly. Cycle sequencing used in MPS generates reads without indel errors. This allows for the non-gapped read-to-read alignments necessary for efficient *de novo* assembly especially by implementing k-mer-based read-to-read indexing using computers with large RAM (e.g., 4TB) and 80 or more cores. Such non-gapped alignments cannot be used for mapping reads to the reference to call indels. Read-to-read non-gaped aligment has previously been implemented in a local *de novo* assembly (Carnevali et al., 2012). Co-barcoding with 10,000 or more barcodes provides substantial localization of reads for efficient and cost-effective assembly of long *de novo* contigs. For example, calling high quality heterozygous variants first (Drmanac et al., 2011) allows the segregation of co-barcoded reads to each parental chromosome for each genomic region having heterozygous positions. For most of the genome, this localization and segregation allows *de novo* assembly of large genomic regions separately for each parental chromosome. Such advanced phased *de novo* assembly would not only provide variant calling unbiased by the reference sequence, it would almost completely prevent calling sequencing errors or mutations introduced by DNA amplification as heterozygous variants. In this approach, the majority of positions in a genome are called with higher accuracy as two hemizygous calls supported by a sufficient number of exclusive parental barcodes. Phased *de novo* assembly using co-barcoded reads from long staggered DNA fragments, especially from ∼50 cells, allows the resolution of variants in complex gene families including hemizygous deletions smaller than an exon. For detection and enumeration of CNVs, counting the number of different barcodes per genomic region separately for each parent allows for a more precise measurement than read counts which are much more prone to various biases. Combined with *de novo* assembly, that defines CNVs by sequence junctions, this advanced LFR process can provide a more complete and accurate genome sequence. Developing advanced co-barcoding and efficient reference unbiased *de novo* assembly of individual human genomes is of critical importance for the broad and efficient implementation of genomic medicine.

### PUTTING IT ALL TOGETHER

**FIGURE 2:**
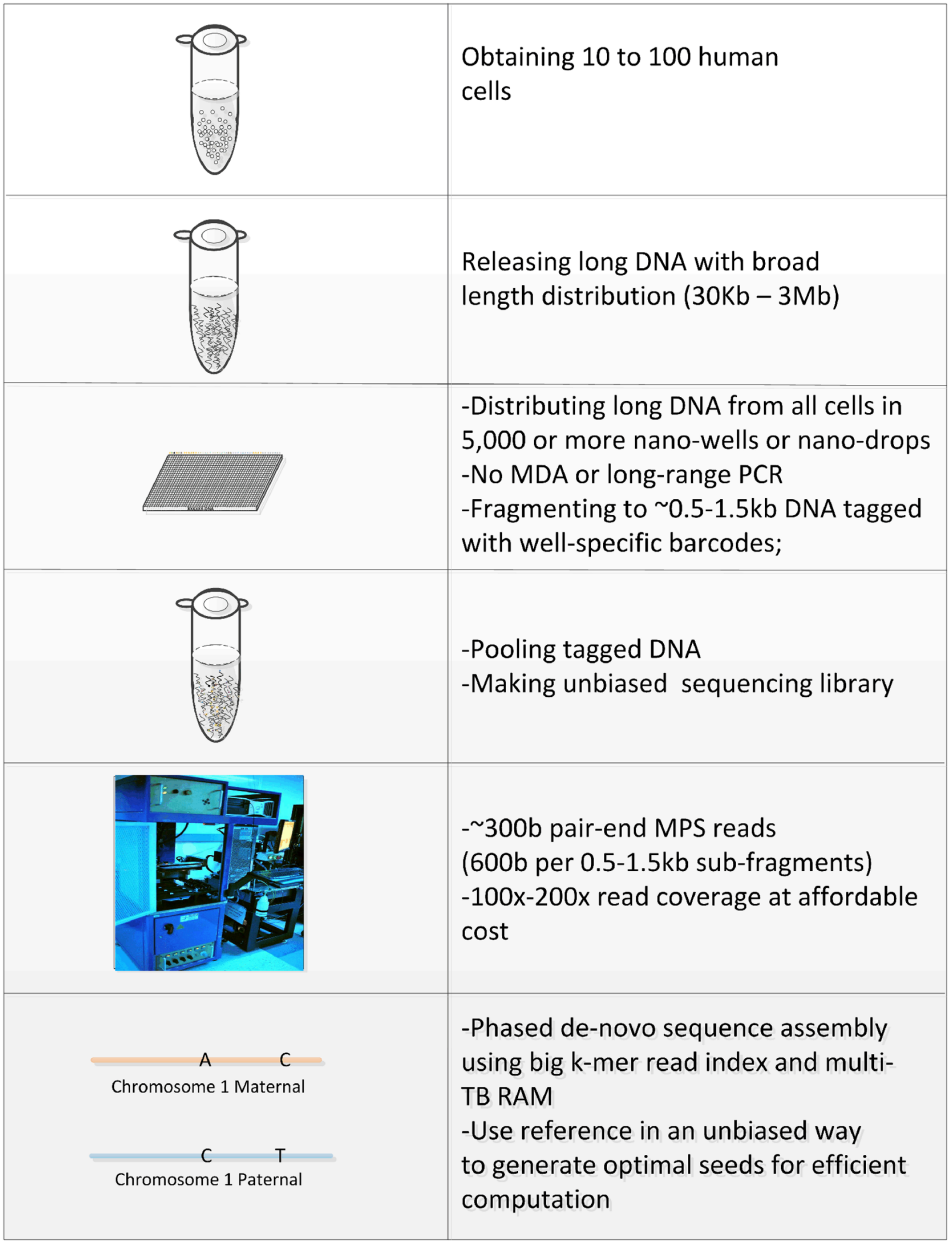
**Integrated “perfect genome” solution.** Our preferred end-to-end proposal for achieving the perfect genome is summarized in this table. The “perfect genome” starts with an input of 10–100 unfixed cells. Cells are lysed and long DNA fragments with a broad length distribution are released and divided into 5,000 or more compartments. Subfragments 500–1,500 bp in length are generated, without long DNA amplification, and labeled with a barcode specific to each compartment (co-barcoded). All compartments are pooled into a single tube and advanced pair-end MPS using 300 base reads is performed, including reading 10 bases or longer barcode sequences. Reference unbiased phased *de novo* assembly of each parental chromosome is performed using k-mer read indexes on large terabyte RAM servers. Obtaining the “perfect genome” from a smaller number of cells (e.g., 10–20) would require maximal optimization of all steps.

In this manuscript we have outlined a proposal for achieving the “perfect genome” sequence using current and yet to be developed technologies. In **Figure 2** we summarize our preferred end-to-end strategy to reach this goal. Almost all parts of the process are currently available but substantial optimization is needed, especially in making representative co-barcoded sequencing libraries in thousands of nano-wells without long DNA amplification and in developing sophisticated phased *de novo* assembly software. Indeed, a recently published paper by Adey et al. (2014) has shown that co-barcoding with ∼10,000 barcodes allows for substantial *de novo* assembly without long DNA amplification. However, due to very low read coverage per long fragment, violating one of the four critical requirements for perfect WGS by co-barcoded reads, a large number of cells (∼500) are required. This results in about 5–10% of a haploid genome per barcode, approximately 10X more than we are proposing. Further, their method is unable to achieve substantial phasing and as such the result is still far from a “perfect genome.”

The most important part of our proposal that is not currently available is a high quality, affordable 300 base MPS paired-end read length (a total of 600 bases read per subfragment). However, given the dramatic improvement in NGS read lengths over the past few years, we believe 300 base reads will be available soon. As discussed, a 10–100 fold low-bias amplification of long DNA coupled with 100 base reads is an alternative solution if 300 base reads are not feasible or cost-effective. It should be noted that while we focused on human genome sequencing for this article, the strategy outlined here is a viable solution for analyzing the genomes of any species.

## CONCLUSION

Improvements to MPS-based NGS continue to advance our ability to efficiently analyze a large number of individual human genomes with high sensitivity and specificity, including separate sequence assembly of parental chromosomes (haplotyping) with recently reported false positive SNV errors of less than 10 per human genome sequenced from a 10-cell sample [Peters et al., under review *Genome Research*]. Co-barcoded reads with at least 5,000 barcodes and advanced phased sequence assembly unbiased by the human reference genome, as proposed here, will further improve accuracy and completeness and allow for the achievement of the “perfect genome.” This type of low cost, high throughput solution promises to enable the efficient and accurate sequence analysis of the entire genome of millions of people as well as from difficult to analyze samples such as micro-biopsies of *in vitro* fertilized embryos and fetal and tumor cells collected from the blood. This ultimate genetic test, when applied broadly, will provide a foundation for genomics-based precision medicine and disease prevention through “genomic healthcare”; enabling a longer and healthier life.

## Conflict of Interest Statement

The authors are employed by Complete Genomics, Inc., a whole human genome sequencing company. Complete Genomics, Inc. is a wholly owned subsidiary of BGI-Shenzhen, a DNA sequencing company. The authors have shares in BGI-Shenzhen. Complete Genomics, Inc. has filed patents on many of the topics discussed in this article.
